# Abrupt Climate Change in an Oscillating World

**DOI:** 10.1038/s41598-018-23377-4

**Published:** 2018-03-22

**Authors:** S. Bathiany, M. Scheffer, E. H. van Nes, M. S. Williamson, T. M. Lenton

**Affiliations:** 1 Department of Environmental Sciences, Wageningen University, Wageningen, The Netherlands; 2 College of Life and Environmental Sciences, University of Exeter, Exeter, UK

## Abstract

The notion that small changes can have large consequences in the climate or ecosystems has become popular as the concept of tipping points. Typically, tipping points are thought to arise from a loss of stability of an equilibrium when external conditions are slowly varied. However, this appealingly simple view puts us on the wrong foot for understanding a range of abrupt transitions in the climate or ecosystems because complex environmental systems are never in equilibrium. In particular, they are forced by diurnal variations, the seasons, Milankovitch cycles and internal climate oscillations. Here we show how abrupt and sometimes even irreversible change may be evoked by even small shifts in the amplitude or time scale of such environmental oscillations. By using model simulations and reconciling evidence from previous studies we illustrate how these phenomena can be relevant for ecosystems and elements of the climate system including terrestrial ecosystems, Arctic sea ice and monsoons. Although the systems we address are very different and span a broad range of time scales, the phenomena can be understood in a common framework that can help clarify and unify the interpretation of abrupt shifts in the Earth system.

## Introduction

**Figure 1:**
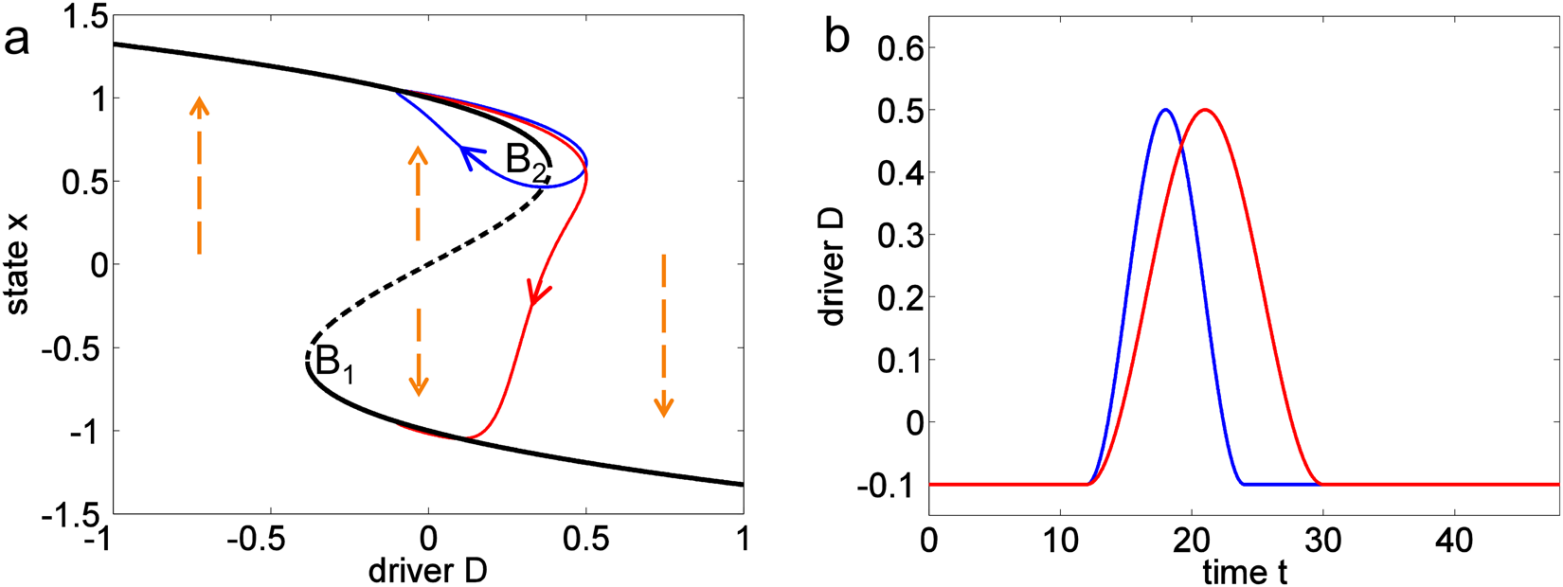
Stable states and trajectories in the example system (Eq. 1). (**a**) The equilibria of state x for constant driver D are shown as black lines (continuous: stable; dashed: unstable). The flow towards a stable state is shown as dashed orange arrows; B_1_ and B_2_ indicate the bifurcation points. (**b**) Time evolution of driver D for two pulses, the red one having a longer period than the blue one. The trajectories of the system that result from these forcings are shown as red and blue curves in (**a**). See Supplementary Information for details on the parameter choices.

The climate and ecosystems usually respond rather linearly to changes in external conditions. However, there are also occasions when at some point a sudden and large shift to a very different state occurs. An important cause of such behavior can be a positive feedback which leads to a self-amplifying change, making the system very sensitive to external conditions. Such feedback-induced threshold behavior is often referred to as a tipping point^1^. If the feedback is very strong, alternative stable states can exist under the same external conditions (Fig. 1a). In models, the parameter values at which the system’s dynamics suddenly changes are called bifurcation points. For example, when the system is driven over a bifurcation point where a current equilibrium ceases to exist, an abrupt and irreversible shift toward a different equilibrium can occur. Tipping points and alternative states exist in many simple models, for example representing ocean circulation^2^, global ice coverage^3,4^, Arctic sea ice^5^, vegetation-atmosphere interaction^6^, terrestrial ecosystems^7^, and the East Asian and Indian monsoons^8,9^. Moreover, there are sometimes also observational indications that multiple states exist in reality^10^, and there is evidence that abrupt shifts have occurred in the past^11–13^.

In the conventional illustration of a tipping point it is assumed that the driver (representing external conditions), varies monotonically and gradually, so that the system stays close to a stable equilibrium (black curve in Fig. 1a). However, many environmental conditions change so rapidly that the system’s state cannot catch up with the changing equilibrium. For example, global warming can drive the Greenland ice sheet into a regime where it is not sustainable anymore^14^. However, the ice sheet can take millennia to melt completely, which gives us a window of “borrowed time”^15^ in which we could save the ice-sheet should the temperature decrease again in time^14^. Figure 1 illustrates the two possibilities of a saved (blue) and a collapsing system (red). As the excursion beyond the threshold takes more time for the red trajectory, the system shifts to the realm of the lower stable branch, while the blue trajectory returns toward its old state.

As natural systems are permanently exposed to environmental change, they are usually not in equilibrium. The external fluctuations that drive them are usually simplified as stochastic, but in reality there often is a dominant timescale as well. This is particularly obvious if the change in external conditions consists in a periodic oscillation, for example the very regular annual and diurnal cycles of solar insolation, and the quasi-periodic changes in the Earth’s orbital parameters. Also, the climate system’s internal variability shows modes with specific frequency ranges, most importantly the El Niño Southern Oscillation (ENSO)^16^.

Such oscillations will affect parts of the climate system or ecosystems that have intrinsic thresholds or bifurcations: a gradual change in the mean, amplitude or frequency of periodic drivers can trigger abrupt change in these systems. In the following, we first illustrate these phenomena with a simple mathematical system. Subsequently, we point out how models and observations indicate the existence of these phenomena in many elements of the Earth system, spanning a vast range of time scales. We illustrate some consequences of this view for monsoon systems, sea ice and terrestrial ecosystems, and argue how a general view on tipping points can contribute to a conceptual understanding of different tipping elements in the Earth system.

## Tipping points in periodically forced systems

To understand the occurrence of tipping points in periodically forced systems, we take a very simple dynamical system as a useful conceptual model example, where the evolution of state x over time t in this system is determined by

1 $$\frac{{\bf{d}}{\bf{x}}}{{\bf{d}}{\bf{t}}}=-{{\bf{x}}}^{3}+{\bf{x}}-{\bf{D}}({\bf{t}})$$

The black lines in Fig. 1a represent this system’s equilibria (dx/dt = 0) for constant D, which we refer to as the equilibrium system. D is an external (prescribed) driver representing environmental conditions, which makes the system non-autonomous (meaning that it explicitly depends on time). In case the driver D changes slowly enough, the system follows a stable equilibrium branch which ends at a bifurcation point. After the driver passes this point the system switches to the remaining stable equilibrium – the case of a classical tipping point.

We now investigate the behavior of the system in response to a driver that oscillates around a mean D_m_ with amplitude D_a_ and period T.

2 $${\bf{D}}({\bf{t}})={{\bf{D}}}_{{\bf{m}}}+{{\bf{D}}}_{{\bf{a}}}\,\cos (\frac{2{\boldsymbol{\pi }}{\bf{t}}}{{\bf{T}}})$$

**Figure 2:**
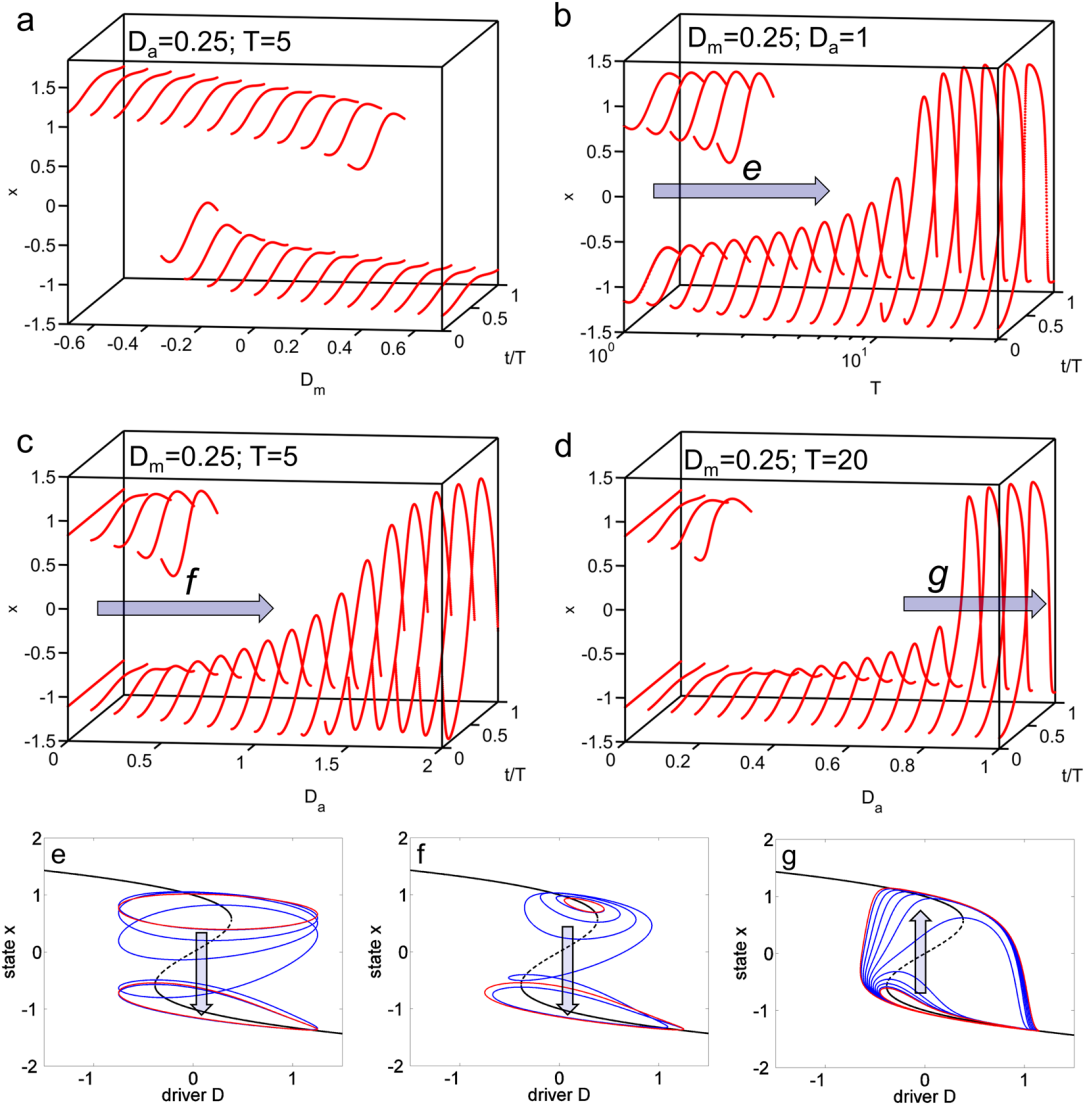
Periodic solutions of the conceptual model (Eqs 1 and 2) in dependence on the driver’s mean D_m_ (**a**), its period T (**b**), and its amplitude D_a_ (**c**,**d**). The blue arrows show the start and end points of linear parameter changes discussed in the text and shown as blue trajectories in (**e**,**f**). The arrows in e-f indicate the overall direction of the change. The two red orbits in each bottom panel are the asymptotic solutions for the parameters at the beginning and end of each transition. See Supplementary Information for details on the parameter choices.

Our conceptual model is hence determined by Eqs 1 and 2, and is well-known as the overdamped limit of the Duffing oscillator. We integrate the system for different sets of the three parameters, D_m_, D_a_ and T, until it reaches a stable attractor in form of a periodic oscillation. Like the driver, the system’s solution is periodic in time with period T (also involving harmonics that can arise close to a bifurcation point)^17,18^. Figure 2 illustrates how the periodic solutions of the system can change when one of the three parameters is varied. Varying D_m_ (Fig. 2a) is similar to the classical scenario of a tipping point, only that the stable solutions oscillate in time: if D_m_ is increased across the bifurcation, the upper branch of stable solutions disappears and the system has to shift to the lower branch. Interestingly, such tipping-point behavior can also be obtained by increasing the driver’s period T or its amplitude D_a_, without any change in D_m_ (Fig. 2b–d). For example, when the driver is fast (small T) the system regularly passes the bifurcation point of the equilibrium system but returns in time to keep the system close to the upper branch. Therefore, there can be two alternative periodic solutions. However, when the driver becomes slower, the system destabilizes and propels itself toward the only remaining solution around the lower branch. Figure 2e illustrates this forced transition as a blue trajectory in the phase space spanned by driver D and state x.

A similar result is obtained when D_a_ is increased (Fig. 2c,f) because the relaxation toward the lower branch becomes too strong. A systematic sampling of the parameter space spanned by D_m_, D_a_ and T shows that D_a_ and T essentially have a very similar effect in the model (Figs S1,S2) as they both reduce the D_m_-range in which multiple solutions occur. When both become large, the dependence on initial conditions is destroyed (Fig. S3). This is due to another type of abrupt transition, which occurs at D_a_ = 0.8 in Fig. 2d. At this point, the system reaches a regime where it makes a full cycle involving both branches of the equilibrium system, flipping up and down between the branches (Fig. 2g). As the system’s response becomes very slow at the bifurcation point of the equilibrium system, the transition to the alternative state occurs after the bifurcation point is reached^19^. Nonetheless, if the driver’s amplitude is large enough, two abrupt shifts occur within each full cycle of the system under constant parameter conditions. When this regime becomes active, the amplitude of the solution increases rapidly with D_a_. In contrast to the bifurcations mentioned previously, such sudden amplitude change is reversible: for intermediate D_a_ in Fig. 2d, there are no multiple solutions because all movement occurs along one continuous stable branch far from the second one above. When D_a_ increases, the driver suddenly encloses both bifurcation points. During a cycle, the system now switches to the upper branch, but falls down again later on the other side. It should be noted that the amplitude change is only a sharp transition if T is sufficiently large (compare Fig. 2c with 2d). Otherwise, the time is too short for the system to get close to the alternative branch to which it is suddenly attracted in a short period during each cycle.

This reminds us that Fig. 2a–d displays only asymptotic solutions where the system ends up after an infinite time. In similarity to tipping points in an equilibrium system, the time it takes do make a transition from one to the other solution (blue trajectories in Fig. 2e–g), depends on the response time of the system. This response time can be idealized as a single relaxation time *τ*. For example, in the equilibrium system given by Eq. 1 and constant D, the relaxation time can be shown to be

3 $${\boldsymbol{\tau }}={\bf{1}}/({\bf{3}}{{\bf{x}}}_{{\bf{eq}}}^{{\bf{2}}}-{\bf{1}}),$$

where x_eq_ is a stable equilibrium and lies on either of the continuous black lines in Fig. 1a.

The ratio between τ and the driver’s period T determines the abruptness of every one of the transitions discussed above. Three regimes can be distinguished: when *T* ≫ *τ*, the system closely follows the stable state of the equilibrium system. This limit case is usually addressed with the classical tipping point concept. When *T* ≪ *τ*, the system cannot follow the rapid forcing at all and remains static. It can then be described with an equivalent time-independent system. In the intermediate regime, where the time scales of the system and driver are similar, irreversible amplitude or timescale-induced shifts can occur.

## Consequences for tipping elements in the Earth system

**Figure 3:**
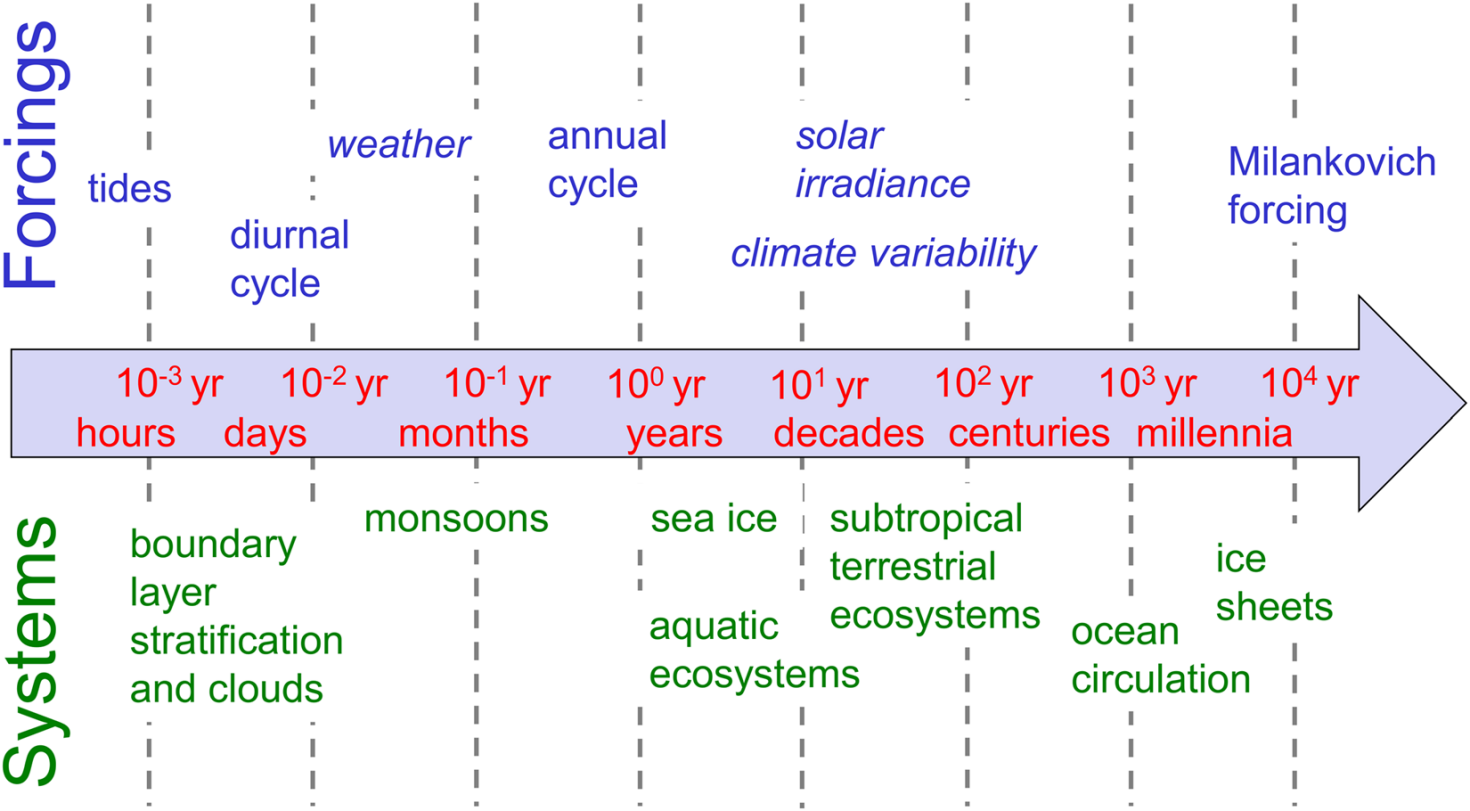
Oscillations in the Earth system that can modulate their amplitude or period, and potentially multistable systems affected by such change. Forcings that are not truly periodic but that have modes of variability on specific time scales are shown in italic font.

Mathematically, bifurcations in time-independent and periodically forced systems are equivalent^20^. However, transitions that are triggered by changes in periodic forcing have not been explicitly included in the concept of climate tipping points which usually build on the simple paradigm of stable equilibria (and their loss of stability at a tipping point)^1,21^. This stands in contrast to the fact that the climate system is interspersed with a number of internal and external oscillations that may cause such transitions on a vast range of time scales. This seems plausible because hardly any oscillation remains the same over time. For example, the seasonal cycle of solar insolation varies due to the Earth’s precession, which in turn is modulated by eccentricity. Similarly, the diurnal insolation cycle depends on the seasons, the Earth’s orbital parameters and its rotation rate. Moreover, modes of internal climate variability often have a distinct range of frequencies, and affect ecosystems and other components of the climate system. Besides these naturally occurring phenomena, the human interference with the climate system is another reason why such oscillations might change. For example, a human impact on the amplitude and frequency of ENSO can be expected^22^. Even the amplitude and timing of the annual cycle can change in many variables when the balance of climate feedbacks shifts under increased greenhouse gas concentrations, for example associated with changes in Arctic cloud cover, or the loss of sea ice^23^. Figure 3 gives a non-exhaustive overview of (quasi-) periodic oscillations and the nonlinear systems they affect. The possibility of tipping points induced by quasi-periodic drivers emerges from a large body of literature on these systems, but is hardly discussed in such process-based studies or in review articles on tipping points.

Starting our overview with small scales, tipping point phenomena have a long tradition in the nonlinear world of fluid dynamics^24,25^. For example, it has lately been found that the structure of the nocturnal atmospheric boundary layer over land can show two alternative regimes^26,27^. The question which regime will be established can crucially depend on the conditions during the evening^28^. On a much larger scale, marine boundary layer clouds show distinct regimes in observations and multiple equilibria in specific models^29,30^. It therefore seems rewarding to study how the diurnal cycle of solar insolation^31,32^ or the annual cycle of sea surface temperatures can affect the long-term structure of the lower atmosphere.

Particularly important for the lives of many people are the abrupt shifts in monsoon systems occurring within the seasonal and orbital cycles. Similarly, the current loss of summer sea ice reminds us that sea ice is nonlinear system that is subject to a marked seasonal cycle. Moreover, multiple equilibria are supposed to exist in several marine and terrestrial ecosystems including lakes^33^, marine sea-grass ecosystems^34^, tropical forests, and drylands^35^. In terrestrial ecosystems, rainfall is often the most important environmental driver, and the longevity and severance of droughts has long-term consequences. We will discuss the cases of monsoons, sea ice and terrestrial ecosystems in more detail below.

Finally, an iconic example of a tipping point is the Atlantic Meridional Overturning Circulation (AMOC) which can show multiple states in models of different complexity^2,36^. Probably related to the AMOC are the quasi-regular Dansgaard-Oeschger events which have been argued to be caused by a regular driver^37^. A subject where the role of periodic drivers has been discussed explicitly are glacial-interglacial cycles. In particular, it has been shown that they can conceptually be understood as forced shifts in a multi-stable landscape^38^. Due to the inertia of the cryosphere and lithosphere, the climate is not in equilibrium with the orbital forcing. Small insolation differences from one orbital cycle to the next can thus cause very different climates^39,40^. Such a conceptual view can also be beneficial for understanding other components of the Earth system and can inspire research questions. We will illustrate this by focusing on three example cases.

## Monsoon systems

Monsoon systems cover a large part of the subtropical land areas and supply many people with water. The most important monsoons occur in India, East Asia, West Africa, Australia and North and South America. The classical view of monsoon systems is that subtropical heat lows in summer induce a low-level circulation from the tropical ocean towards higher latitudes that supplies the subtropical land with moisture^41^. Due to geographical differences between the monsoon regions, each monsoon system is usually studied on its own. However, observations from several monsoon systems show an intriguing asymmetry in the annual cycle of precipitation: while the onset of the monsoon rainfall in spring tends to occur rather abruptly, its retreat in autumn occurs much more gradually. Abrupt monsoon onsets have been observed in West Africa^42^, the southwest Indian coast^43^, and in East Asia, where several abrupt stages of monsoon onset have been identified^44^. This behavior stands in contrast to the gradual and symmetric annual cycle of solar isolation that drives the monsoon evolution.

There are many potential reasons for abrupt monsoon onsets. First, meteorological theory predicts that switches from a zonal to a meridional flow can occur due to hydrodynamic instabilities when the atmosphere is sufficiently heated away from the equator^45,46^. In particular, the mechanism has been found to operate in a regional model of West African monsoon climate^47^ and is supported by observations^48^. Second, air-sea interactions involving wind, evaporation, clouds and moisture advection can provide positive feedbacks which can rapidly enhance the monsoon after its onset^49,50^. One important positive feedback results from the advection of moisture by the low-level monsoon flow. When this moisture condenses over land, the latent heat release further enhances the circulation. This feedback is at the heart of the monsoon model by^51^ that produces abrupt monsoon shifts, and whose simplified version^8^ we will apply below. Third, the distribution of land surface properties such as albedo and orography modulate the monsoon onset^52–54^. Fourth, the seasonal cycle is superimposed by faster oscillations, causing periods of rapid change^42,55^. Such oscillations can make the monsoon onset more abrupt because they tend to be phase-locked to the annual cycle^56,57^.

Despite the complexity and geographical differences between the monsoon systems, the widespread existence of sudden monsoon onsets invites describing them within the common concept of tipping points. In addition, the annual cycle of solar insolation is a cyclic driver of these systems. One could therefore speculate that monsoons are periodically driven bistable systems. As the monsoon circulation adjusts very quickly to its boundary conditions it would be best described as a system with a very small ratio *τ*/*T*, similar to the example system in Fig. 2d and g. One could therefore think of a typical annual cycle as a complete loop over both branches of the bistable system (the large amplitude solutions in Fig. 2d). It then follows that a small shift in external conditions could bring the system into a regime where the bifurcation point of the monsoon onset is not reached anymore in a particular year. Indeed, past abrupt transitions between episodes of strong and weakened (“failed”) East Asian monsoon have been identified in Chinese cave records^13^. These switches occur due to the oscillations of the Earth’s orbit that modulate summer insolation on the northern hemisphere. In this regard, the behavior of monsoons within a year and on timescales of hundred thousands of years is remarkably similar.

## Model example: sudden onset and failure of monsoons

To conceptualise the link between abrupt monsoon transitions on different time scales we use the simple model of Levermann, *et al*.^8^ which describes an idealized summer monsoon that can switch between an “on” and “off” state (see Supplementary Information). The mechanism responsible for the abrupt behavior in the model is the moisture-advection feedback. It has recently been challenged that this feedback would be strong enough to support such nonlinearity^58–60^. While the underlying mechanism is hence uncertain, it is clear that abrupt monsoon switches both in the seasonal cycle and in response to orbital forcing do occur. By applying the tipping point framework we speculate that a common mechanism is behind the jumps within the annual cycle and the abrupt transitions on millennial time scales. While the Levermann model suggests the moisture-advection feedback as the cause, any other positive feedback would essentially yield the same qualitative behaviour.

**Figure 4:**
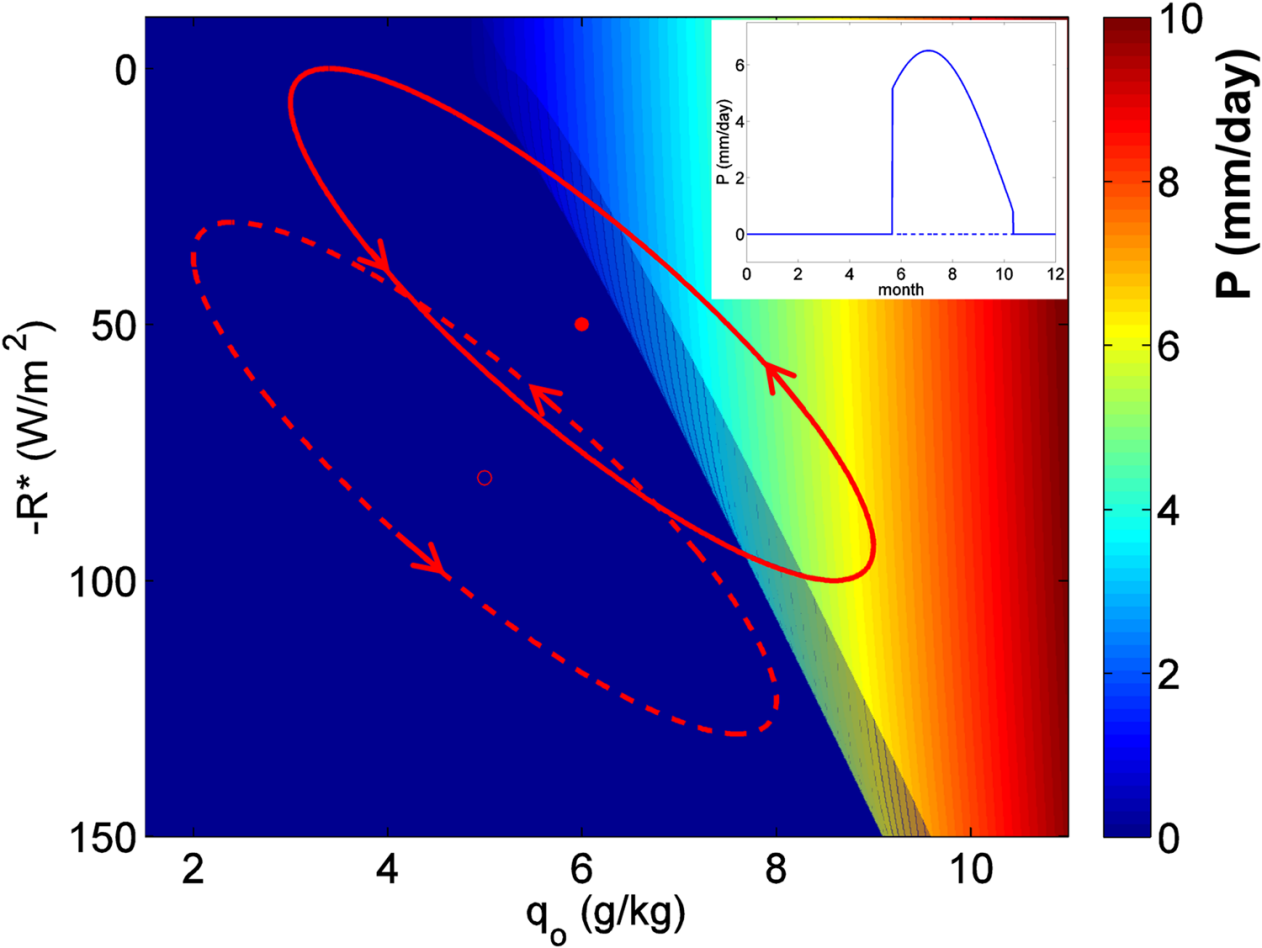
Summer rainfall in mm/day in the monsoon model in dependence on oceanic specific moisture and net atmospheric radiation. The two red orbits show an annual cycle of the two parameters where the annual mean state is indicated with a dot. The resulting annual cycle of precipitation of each orbit is shown in the inset with continuous (dashed) lines for a situation where conditions for a monsoon onset are met (not met) in summer.

The most important parameters that determine the model’s state are the atmospheric humidity over the ocean q_o_ and the radiative balance of the atmospheric column over land R. Without any alterations to the model we generalize R here to also include the sensible heat flux at the Earth’s surface and call the resulting quantity R*. Figure 4 shows the precipitation P associated with the summer monsoon as a result of q_o_ and R*. The threshold that separates the “on” and “off” states can be seen as a sharp boundary between the blue area (no monsoon rainfall) and other colors (monsoon rainfall of different magnitude).

We now impose an annual cycle on the parameters q_o_ and R*. The model is only valid for summer conditions and cannot describe any precipitation not associated with the summer monsoon. We therefore set P to zero in other seasons, knowing that no monsoon rainfall occurs during that time. In order to obtain an asymmetric annual cycle with an abrupt onset like in observations, we apply two strategies:

1. We follow the approach by^9^ by introducing a small bistable regime at the critical threshold (the shaded region with a width of 0.5 g/kg at the boundary).
2. We introduce a phase lag of one month between the two cyclic drivers, making oceanic moisture q_o_ lag the seasonal cycle in R*. This approach may be justified by the different memories of ocean and atmosphere: while the atmosphere can adjust to the solar insolation within days, the oceanic temperature which controls specific humidity lags the insolation cycle by many weeks. The resulting annual cycle of q_o_ and R* is shown as a red orbit in the parameter plane of Fig. 4, rotating counter-clockwise.

The annual cycle of rainfall that results from the trajectory of q_o_ and R* is shown in the inset of Fig. 4. As soon as the “off” state disappears in early summer at the right border of the shaded bistable region, an abrupt monsoon onset occurs. Later in the summer, R* and q_o_ decline and rainfall decreases with them, until it drops to zero when the “on” state disappears at the left border of the shaded bistable region. An alternative situation with a different mean of q_o_ and R* is shown by the dashed orbit and dashed annual precipitation cycle, where precipitation is zero during the whole year. The transition from the regime where a monsoon circulation establishes each year to the regime where it does not establish constitutes a generalized tipping point. The monsoon collapse occurs when the threshold value of q_o_ and R* is not reached anymore in summer.

## Arctic sea ice

It has been hypothesized whether global warming can cause abrupt and irreversible sea-ice loss in the Arctic^61^. The most important argument for this idea is the positive feedback between surface albedo and sea-ice loss (ice-albedo feedback). It has been found that the existence of bifurcations in sea ice models depends on the description of spatial differences and the seasonal cycle^62,63^. While future ice loss is reversible in comprehensive models, these models also indicate that Arctic winter sea ice can be very sensitive to warming^64^. Moreover, a comprehensive model shows three alternative stable sea-ice states on a completely land-free Earth^65,66^. One of these states is a globally ice-covered planet, resembling the “snowball” conditions that occurred during the Neoproterozoic. This state also occurs in at least one other complex model under present-day boundary conditions^67^.

Hence, it is plausible that tipping points in sea ice have occurred in the Earth’s past, triggered by changes in the continental configuration, Milankovitch forcing, or the seasonal insolation cycle. In this regard, Eisenman^68^ provides an insightful parameter analysis of a simple Arctic sea ice model that is driven by the annual cycle of solar and terrestrial radiation. Small variations in these fluxes can lead to abrupt changes in the annual cycle of sea ice. Compared to the previous systems, Arctic sea ice and the surface ocean constitute a slow system that is not in equilibrium with the annual cycle of insolation. This can be seen from the fact that the annual cycle of Arctic sea ice volume lags the insolation cycle by approx. three months. The analysis by Eisenman^68^ was performed with a model that already included the annual cycle of short- and long-wave insolation. The concept of tipping points in periodically forced systems suggests that the bifurcations in the model originate from multiple states under certain fixed values of insolation, as the following model results show.

## Model example: abrupt Arctic winter sea ice loss

**Figure 5:**
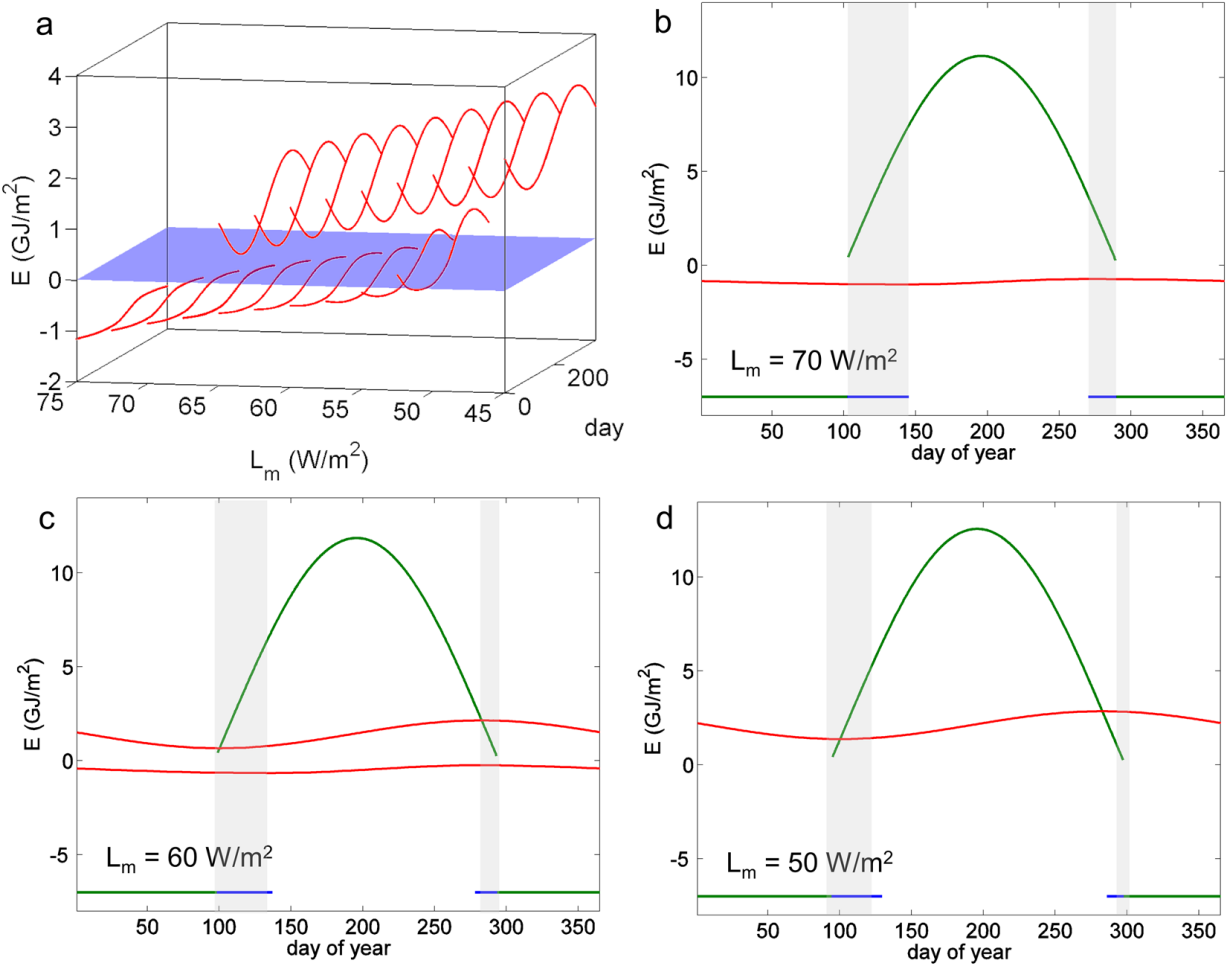
Solutions of the Arctic sea ice model in dependence on the mean long-wave radiation balance L_m_. (**a**) Full range of periodic solutions between L_m_ = 75 and 45 W/m^2^. The blue surface demarcates between sea ice (E < 0) and open ocean (E > 0). Panels b–d show the solutions for three different values of L_m_ in red; green and blue lines show equilibria for fixed solar and terrestrial irradiation representing a certain day of year when starting from very high (green) or very low (blue) E. The ranges where two equilibria are possible for the same irradiation are shown as blue shaded areas.

The model by Eisenman^68^ calculates an energy balance for a well-mixed box of ocean water, covered with ice of a single thickness (see Supplementary Information). When ice is present, the enthalpy E is negative and proportional to the ice thickness. In the absence of ice, E is positive and proportional to the ocean temperature. The bifurcation parameter L_m_ represents the annual mean outgoing long-wave radiation budget at the surface (not including temperature feedbacks) and becomes smaller when the climate warms. For values of L_m_ between approx. 65 W/m^2^ (which roughly represents the present-day climate) and 50 W/m^2^, two stable solutions can be found: a seasonally ice-covered ocean and an ice-free ocean (Fig. 5a).

The solutions in Fig. 5 are periodic in time because of the prescribed annual cycle in the long-wave budget L, and downwelling short-wave radiation, S. Figure 5b–d again displays the annual cycle for three distinct values of L_m_ (red lines). In addition, we show to what state the sea-ice system would evolve if there was no annual cycle (green and blue lines). To this end, we fix S and L to the values corresponding to a particular day in a year and integrate the model forward in time, starting from large positive and negative E. For most days in the year, only one asymptotic solution exists. For example, under permanent summer conditions, all ice melts away and the ocean becomes so warm that the outgoing long-wave radiation balances the absorbed radiation (Fig. 5, green line). Under permanent winter conditions, the ice grows to an infinite thickness, because there is no negative feedback in the model to limit the ice growth. In such cases of failed convergence we artificially set the enthalpy to −7 GJ/m^2^ (corresponding to a thickness of approx. 23 m). As the ice-ocean system cannot fully adjust to the fast insolation changes in the course of a year, the amplitude of its annual cycle is very small compared to the difference of its steady state solutions. The solutions overlap only during a short period in spring and an even shorter period in autumn, in the rest of the year only one solution exists. Nonetheless, the existence of multiple solutions still carries over to the periodically forced system for a certain parameter regime.

The bifurcations in this default model (Fig. 5a) can thus be understood from the change of the asymptotic solutions with L_m_. In a cold climate, the summer is too short and insolation is too low to melt enough ice within one summer; a perennial ice cover is therefore the only solution of the periodically forced system. Under global warming (decreasing L_m_), the asymptotic solution moves to larger E and the melting season becomes longer (Fig. 5c); this is where the periodically forced system has multiple solutions, balancing between the cold and warm attractors. Finally, the winter becomes too short for the formation of sea ice thick enough to survive the following summer, and the ice-free solution is the only one that remains (Fig. 5d).

In conclusion, the fact that a certain comprehensive model does not show multiple solutions under the same external conditions does not automatically suggest that tipping points are not possible. This has two reasons: First, tipping points can remain hidden in a parameter range related to periodic forces that is not explored when varying one parameter (such as CO_2_) alone. As Eisenman’s single column model above shows, the existence of multiple equilibria under fixed seasonal conditions in a model is a ‘smoking gun’ pointing to a strong positive feedback. Such a feedback indicates that the system is prone to tipping points even if the multistability is not apparent in the seasonally forced (non-autonomous) system. Changes in the insolation forcing (for instance due to changes in the annual cycle of cloud cover) can hence bring the system into a regime where abrupt changes are possible. The same can occur due to changes in the intrinsic relaxation time of the system, for instance related to the fact that the growth rate of sea ice depends on its thickness and hence the background climate^69,70^. Second, abrupt shifts can also occur in systems without multiple states. Specifically, the freezing point of water acts as a natural threshold for the formation of sea ice, making ice cover very sensitive on temperature in a certain regime and prone to reversible abrupt shifts^64^. The quasi-periodic changes in insolation related to Milankovitch forcing might therefore switch the Arctic back and forth between an ice-free and a seasonal ice regime (in analogy to the monsoon switches outlined above). A similar switch-mechanism determining the growth and melt of ice sheets has also been discussed as an explanation of ice ages during the Pleistocene^71^.

## Terrestrial ecosystems

Multiple equilibria are thought to exist in several marine and terrestrial ecosystems including lakes^33^, marine sea grass ecosystems^34^, tropical forests, and drylands^35^. As an example we consider the bistability of tropical forest versus savannah. For instance, satellite observations across continents show a multimodality in the probability distribution of tree cover under the same precipitation regime^10,72^. The likely cause is the competition between tropical grasses and trees and their different vulnerability to fire^73,74^: in a savannah ecosystem, fire occurs frequently and as grasses can quickly re-establish burned areas they outcompete the slower-growing tree saplings. In contrast, where a forest has established, fire is less likely to occur and the high tree cover can persist at the expense of grasses. Dynamic vegetation models indeed indicate that large subtropical land areas fall into a precipitation regime where several stable states are possible^75,76^.

However, precipitation is not constant but varies on multi-annual time scales. In the tropics, rainfall over land is often controlled by the magnitude and distribution of sea surface temperature (SST). Tropical SSTs show characteristic modes of variability like the El Nino Southern Oscillation (ENSO), Pacific decadal oscillation (PDO) and Atlantic multidecadal oscillation (AMO) which affect tropical rainfall. For example, high Atlantic SSTs can lead to severe droughts in the Amazon like those observed in recent years^77^, and changes in ENSO activity strongly affect Amazon rainfall and vegetation^78^. Proxy-based reconstructions suggest that ENSO amplitude was low during the mid-Holocene, and relatively high in recent decades^79^. Moreover, tree rings indicate that ENSO amplitude and frequency have changed substantially on decadal to centennial time scales during the last 1,000 years^16,80^. The time scales of such changes are on the same order of magnitude than the time scale of tree growth which suggests the possibility that terrestrial tipping points can be triggered by changes in ENSO variability^81^. It is important in this context that a dieback caused by a large-scale drought can occur within a much shorter time than regrowth – in contrast to our example model above, the system is not symmetric. This time scale asymmetry suggests that forests are not safe from a drought-induced collapse even if the long-term mean rainfall is very large.

Moreover, precipitation is not strictly external, but interacts with vegetation. Such atmosphere-vegetation interaction is believed to be particularly large in Northern Africa due to positive surface-albedo and water cycle feedbacks^82,83^. It has therefore been investigated if these feedbacks can give rise to multiple equilibria and whether the end of the Green Sahara some 5,500 years ago can be interpreted as a tipping point^6,12,84^. Moreover, rainfall in Northern Africa shows large decadal variations. Climate models show that these variations are caused by SST changes in several ocean basins and that the Sahel drought of the 1970s and early 1980s was caused by such modes of natural climate variability^85,86^. The time scale of these inter-annual fluctuations is of similar magnitude than the time scale of responses in grass coverage in such drylands. It is therefore plausible that rainfall fluctuations can lead to locally amplified shifts between a desert and a grass-covered state (representing a case like Fig. 2c and f).

Building on a conceptual model by Brovkin, *et al*.^6^, Zeng, *et al*.^87^ showed such abrupt shifts in a vegetation model with oscillating precipitation forcing. We reproduce their result in our Fig. S4 as another explicit model example (see Supplementary Information for model description). Despite the model differences, the results are directly comparable to our conceptual model above (Fig. 2c,d). At small amplitude, two alternative vegetation states exist. As soon as the amplitude becomes large enough, a bifurcation occurs, leading to an irreversible vegetation increase when starting from a desert state as an initial condition. At even larger amplitude, the rainfall variations now cover both equilibria and the mean vegetation cover drops to an intermediate average state while having much larger amplitude. Hence, a small change in the amplitude of rainfall fluctuations can result in a vegetation collapse or increase. As in the generic conceptual model above, the abruptness of the second (reversible) type of the transition depends on the timescale of the forcing relative to the relaxation time of the system (top versus bottom panel).

## Toward a more general view on tipping points

For the sake of simplicity, our example models are a very idealized representation of reality. For example, alternative equilibria are arguably less likely or at least harder to find in more complex models^88^. Also, low-order models can often be misleading when spatial dimensions come into play. Taking space into account can often suppress multiple equilibria, but can also lead to new critical phenomena like pattern formation^35^. Despite the relevance of abrupt shifts in the Earth system, the connection between regime transitions in spatially extended systems (like the onset of turbulence in fluids)^24^ and bifurcations in ordinary differential equations is not straightforward^25^, and neither is such theoretical knowledge well applied to the specific physical systems mentioned above. Nonetheless, nonlinearities and critical thresholds are arguably present in all Earth system components, providing a basis for abrupt shifts. At the same time, other yet unrecognized tipping points could exist in the Earth system.

In more realistic systems the drivers also have a more continuous spectrum. In models, the fast time scales in such spectra are often described by stochastic terms. The amplitude and period of the periodic oscillations we assumed above can be seen as an analogy for the typical magnitude and duration of random external fluctuations. Small changes in these statistical properties can lead to large and sometimes counter-intuitive changes in the distribution of a system’s state^89,90^. In this regard, the concept of tipping points triggered by changes in periodic forcings is one representation of the fundamental question how vulnerable components of the Earth system are to environmental change. Theoretical progress has been made in recent decades to generalise concepts from bifurcation theory, for example by defining pullback attractors for deterministic non-autonomous systems^91^, and random attractors for stochastic systems^92,93^. Unfortunately, such theoretical insights do not always feed back to our knowledge on tipping points in the Earth system.

One particular challenge in this regard is to investigate the applicability of statistical stability indicators that could provide an early warning of abrupt change. In climate research, this concept has mostly been explored in the static context of classical tipping points^94–96^. In the case of the systems above, classical indicators like rising variance and autocorrelation are not necessarily the most useful approach^18,97^. It is therefore important to explore alternative approaches to early warnings of abrupt change. Although conceptual models cannot replace studies based on observations and process-based modelling, they can help raise questions for future research. For example, it may be beneficial to compare abrupt monsoon changes on different time scales and in different regions: Are there common mechanisms behind the abrupt onsets in different monsoon systems on the planet? Are these mechanisms also responsible for abrupt monsoon shifts in palaeorecords? Is the expectation to see early warning signals before the occurrence of a certain shift supported by current process understanding? Will stronger El Niño events have irreversible consequences for tropical forest and savannah ecosystems? Was sea ice involved in a tipping point in the Earth’s history, and what was the role of periodic forcings? To explore such questions, it would be beneficial to further develop models of low to intermediate complexity in addition to existing Earth system models. Such a model hierarchy is most established in the case of sea ice models, but patchy in the case of ecosystems, and lacking in the case of monsoons. A suite of models that are easy to use would also better connect research attempts to understand different monsoon systems.

On top of that, studies on isolated tipping elements may even not be sufficient, as different Earth system components are linked in their stability behaviour and thus do not act in isolation. For example, sea-ice loss and the meridional ocean circulation affect each other^65,66,98^, and transitions between vegetation states depend on the global background climate involving the ice sheets^84^ and the Atlantic overturning circulation^84,99^. In light of the large uncertainties, the candidates and suspected mechanisms of tipping points will certainly evolve with new knowledge. As the Earth system is interspersed with feedbacks and natural thresholds, and features oscillations and environmental fluctuations on a vast range of time scales, it seems plausible that non-equilibrium tipping points have already occurred in the past and could occur again in the future.

### Data availability

All analysed data can be produced with the simple models as explained in the Supplementary Information, and can also be obtained from the lead author (S.B.).

## Electronic supplementary material

Supplementary Information
