# Supplementary material for: Abrupt Climate Change in an Oscillating World
Source: Sci Rep. 2018 Mar 22;8:5040. doi: 10.1038/s41598-018-23377-4 (PMC5864911; doi:10.1038/s41598-018-23377-4)
Supplement: Supplementary file 1 — Supplementary Information [file 41598_2018_23377_MOESM1_ESM.pdf]

Abrupt Climate Change in an Oscillating World

S. Bathiany, M. Scheffer, E. H. van Nes, M. S. Williamson, T. M. Lenton

Supplementary Information

Overdamped Duffing oscillator

As a conceptual model for illustratory purposes we use the overdamped Duffing oscillator given by Eq. 1 and Eq. 2 in the main text and repeated here:

$$\frac{dx}{dt} = -x^3 + x - D(t) \qquad \text{(Eq. 1)}$$

$$D(t) = D_m + D_a \cos\left(\frac{2\pi t}{T}\right) \qquad \text{(Eq. 2)}$$

To perform the two cases of a saved and collapsing system (Fig. 1) we first run the system into equilibrium for constant D = -0.1. Thereafter, we let D make one single cycle with the following parameters:

saved system (blue trajectory): D_m = 0.2, D_a = -0.3, T = 12.

collapsing system (red trajectory): D_m = 0.2, D_a = -0.3, T = 18.

For the three transient simulations shown in Fig. 2e-g, we first let the model state x converge to its periodic solution, and then vary the parameters in the following way:

Fig. 2e: D_m = 0.25, D_a = 1, T changes from 2 to 5 within 5 cycles.

Fig. 2f: D_m = 0.25, D_a changes from 0.2 to 1 within 5 cycles, T = 5.

Fig. 2g: D_m = 0.25, D_a changes from 0.7 to 0.9 within 10 cycles, T = 20.

In all above cases, we solve the system numerically with a Runge-Kutta scheme of fourth order, using 50.000 time steps per cycle.

Monsoon model

The monsoon model by Levermann et al.[8] describes the energy and water balance of the atmospheric column over land. The monsoon flow from the ocean advects moisture over land which then condensates in the rising air. The condensational heat is radiated to space.

$$LP - \varepsilon C_p W \Delta T + R = 0 \qquad \text{(Eq. S1)}$$

$$\varepsilon W \rho (q_o - q_L) - P = 0 \qquad \text{(Eq. S2)}$$

$$W = \alpha \Delta T \qquad \text{(Eq. S3)}$$

$$P = \beta (q_L - q_L^0) \qquad \text{(Eq. S4)}$$

Eq. S1 describes the balance between condensational heat, temperature advection and radiation in the atmospheric column. Eq. S2 describes the steady state water balance (moisture convergence = condensation). Eq. S3 describes the momentum equation (temperature gradient drives circulation). Eq. S4 sets a minimum moisture threshold below which no precipitation is possible (monsoon failure). The four equations can be combined to yield one algebraic equation that is cubic in P and therefore has three solutions. Two of these solutions however, are discarded as unphysical due to a wrong direction in the circulation ($W<0$) or negative precipitation ($P<0$)[8,9]. The remaining solution describes the “on” state of the monsoon. In a parameter regime where only imaginary or unphysical solutions exist, the monsoon is thought to be in its “off” state, and precipitation is then set to 0.

The model is only valid for summer conditions and cannot describe any precipitation not associated with the summer monsoon. We therefore set P to zero in other seasons, knowing that no monsoon rainfall occurs during that time. Moreover, the model ignores the sensible heat flux at the surface, SH, because it is small in summer. Here we do not make this assumption but lump together SH and R to become $R^* = R+SH$. The equations then do not change, except that R is replaced by R* with a different meaning. R* and the specific moisture over the ocean, q_o, are the two external parameters that we assume to oscillate with a period of one year.

In order to obtain an asymmetric annual cycle with an abrupt onset like in observations, we apply two strategies:

1. We follow the approach by Schewe et al.[9] by introducing a small bistable regime at the critical threshold (the shaded region with a width of 0.5 g/kg at the boundary).

2. We introduce a phase lag of one month between the two cyclic drivers, making oceanic moisture q_o lag the seasonal cycle in R*. This approach may be justified by the different memories of ocean and atmosphere: while the atmosphere can adjust to the solar insolation within days, the oceanic temperature which controls specific humidity lags the insolation cycle by many weeks.

Parameters and variables

P	monsoon precipitation (mm/day)	
W	circulation strength	
ΔT	temperature difference between land and ocean	
R	radiative budget of the atmospheric column	
q_o	specific humidity over the ocean	
q_L	specific humidity over land	
L	latent heat of condensation	$2.6\ 10^6$ J/kg

ε	aspect ratio between vertical and horizontal scale	6.7 10^{-2}
C_p	heat capacity of air at constant pressure	1295 J/m^3K
q_L^0	minimum specific moisture over land	4.73 g/kg
ρ	low-level air density	1 kg/m^3
α	efficiency of meridional circulation (W/ΔT)	3 m/sK
β	condensation efficiency	0.0205 kg/m^2s

Arctic sea ice model

The Arctic sea ice model by Eisenman and Wettlaufer[5] in the version of Eisenman[68] calculates an energy balance for a well-mixed box of ocean water, covered with ice of a single thickness. The incoming short-wave and long-wave radiation at the surface are prescribed as harmonic oscillations. The only dynamic state-variable in the model is enthalpy E. When ice is present, the enthalpy is negative and proportional to the ice thickness h_i. In the absence of ice, E is proportional to the ocean temperature T_o:

$$E \equiv \begin{cases} -L_i h_i, \ E < 0 \ [sea\ ice] \\ c_o H_o (T_o - T_m), \ E \geq 0 \ [open\ water] \end{cases} \quad \text{(Eq. S5)}$$

L_i is the latent heat of melting/freezing, c_o the water's heat capacity, H_o the mixed layer depth, and T_m the mixed layer temperature.

The evolution equation of the model is a simple energy budget:

$$\frac{dE}{dt} = A - BT \quad \text{(Eq. S6)}$$

where B*T is the linearized blackbody emission and A is the sum of all temperature-independent fluxes:

$$A = \left(\alpha + \frac{\Delta_\alpha}{2}\tanh\left(\frac{E}{L_i h_\alpha}\right)\right)\left(S_m - S_a \cos 2\pi \frac{t}{P}\right) - \left[L_m + L_a \cos 2\pi\left(\frac{t-\Phi}{P}\right)\right] \quad \text{(Eq. S7)}$$

The bifurcation parameter L_m represents the annual mean outgoing long-wave radiation budget at the surface (not including temperature feedbacks) and becomes smaller when the climate warms. For values of L_m between approx. 65 W/m^2 (which roughly represents the present-day climate) and 50 W/m^2, two stable solutions can be found: a seasonally ice-covered ocean and an ice-free ocean (Fig. 5a).

The temperature T in Eq. S6 represents the difference between the surface temperature and the freezing temperature of ice. In the presence of sea ice, the temperature distribution within the ice layer is assumed to be linear. During melting, T is at the melting point:

$$T = \begin{cases} \frac{E}{c_o H_o}, \ E \geq 0 \quad [open\ ocean] \\ 0, \ E < 0, A \geq 0 \ [melting\ surface] \\ \frac{A}{B}(1 - \frac{\zeta}{E})^{-1}, \ E < 0, A < 0 \ [frozen\ surface] \end{cases} \quad \text{(Eq. S8)}$$

In our experiments without annual cycle, we set time t in Eq. S7 to a constant day of the year, but still integrate Eq. S6 over time. Under permanent winter conditions, sea ice then grows to an infinite thickness. In such cases we limit E to an arbitrary lower bound of -7 GJ/m^2 (corresponding to a thickness of approx. 23 m).

Parameters and variables

E	enthalpy (J/m^2)	
A	absorbed short- and long-wave radiation	
T	ice temperature	
T_m	mixed-layer temperature	
L_m	annual mean long-wave radiation balance	
t	time	
h_i	ice thickness	
L_a	amplitude of long-wave radiation balance	41 W/m^2
S_m	mean downwelling surface short-wave radiation	100 W/m^2
S_a	amplitude of downwelling surface short-wave radiation	150 W/m^2
P	Period of insolation	1 year
B	linearized long-wave emittance according to Stefan-Boltzman's law	2.83 J/m^{2}sK
Φ	phase lag between short-wave and long-wave annual cycle	0.15
ζ	thermodynamic scale thickness	0.7
α	mean surface albedo	0.56
Δ_α	albedo difference between ice and water	0.48
L_i	latent heat of melting	3 10^8 J/kg
h_α		0.5
c_oH_o		2 10^8

Vegetation model

The model by Zeng et al.[87] describes the interaction of vegetation V and precipitation P in North Africa. V can be interpreted as the fraction of leaves relative to their maximum coverage. The model is a non-dimensionalised version of earlier, very similar concept models[6]. The only dynamic equation describes the evolution of V, based on a relaxation approach:

$$\frac{dV}{dt} = \frac{P^a}{P^a+1} - V \qquad \text{(Eq. S9)}$$

As rainfall P adjusts much faster to changes in V than vice versa, P is determined by an algebraic equation.

$$P = max\left[P_1 + \mu V + F_0 \sin\left(\frac{2\pi}{T}t\right), 0\right] \qquad \text{(Eq. S10)}$$

The sinusoidal signal captures the low-frequency fluctuations that arise from sea surface temperatures in an idealised way.

Parameters and variables

V	vegetation fraction	
P	precipitation	
t	time	
μ	feedback parameter describing the effect of vegetation on rainfall	2

P_1	background precipitation parameter	0.2
a	parameter describing the nonlinear shape of the vegetation's response function	4
T	period of rainfall fluctuations	
F_0	amplitude of rainfall fluctuations	

Supplementary Figures

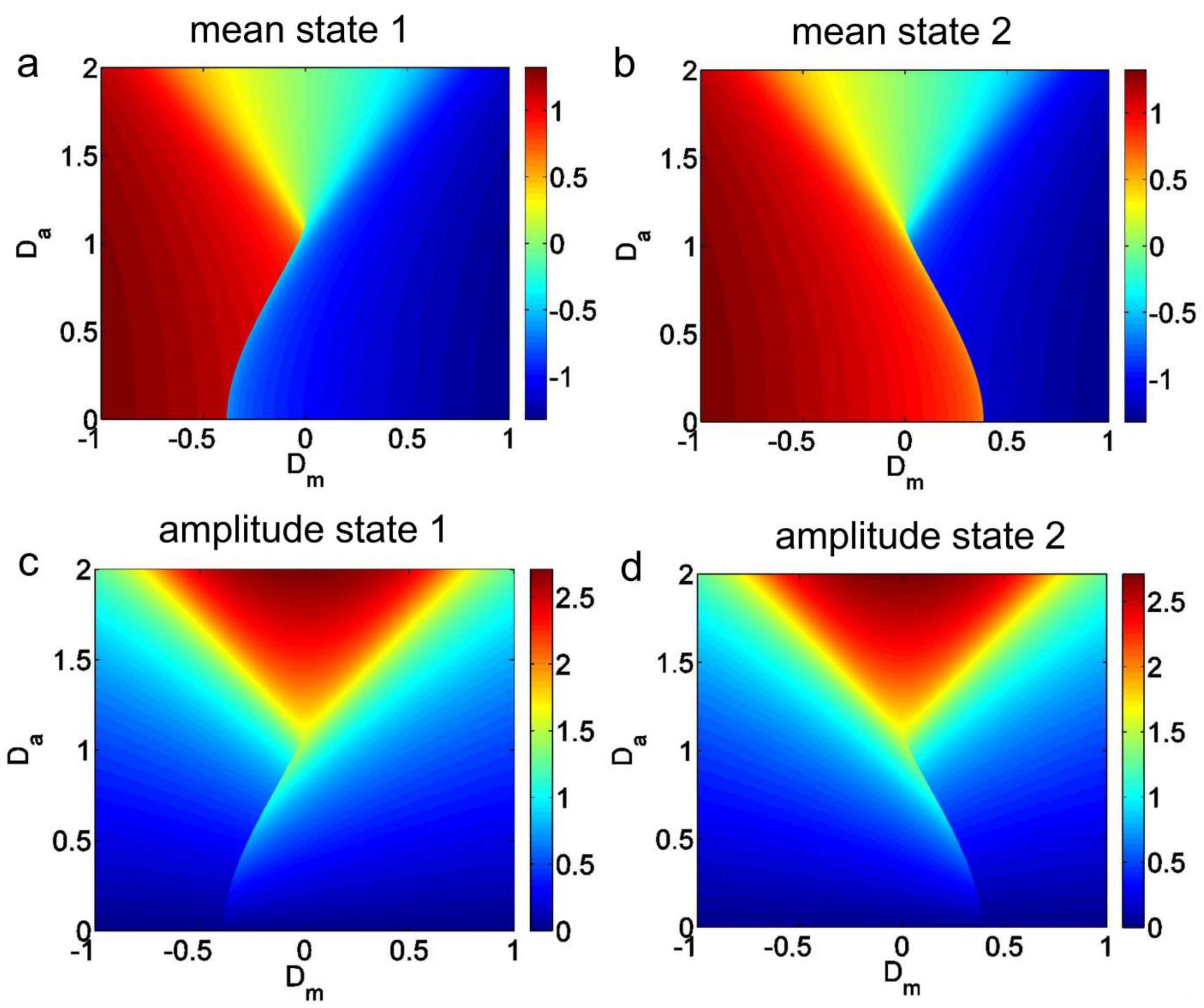

Fig. S1. Behavior of the overdamped Duffing oscillator for different time mean D_m and amplitude D_a of driver D. a) time mean state when initialized near the lower equilibrium, b) time mean state when initialized near the upper equilibrium, c) state amplitude when initialized near the lower equilibrium, d) state amplitude when initialized near the upper equilibrium.

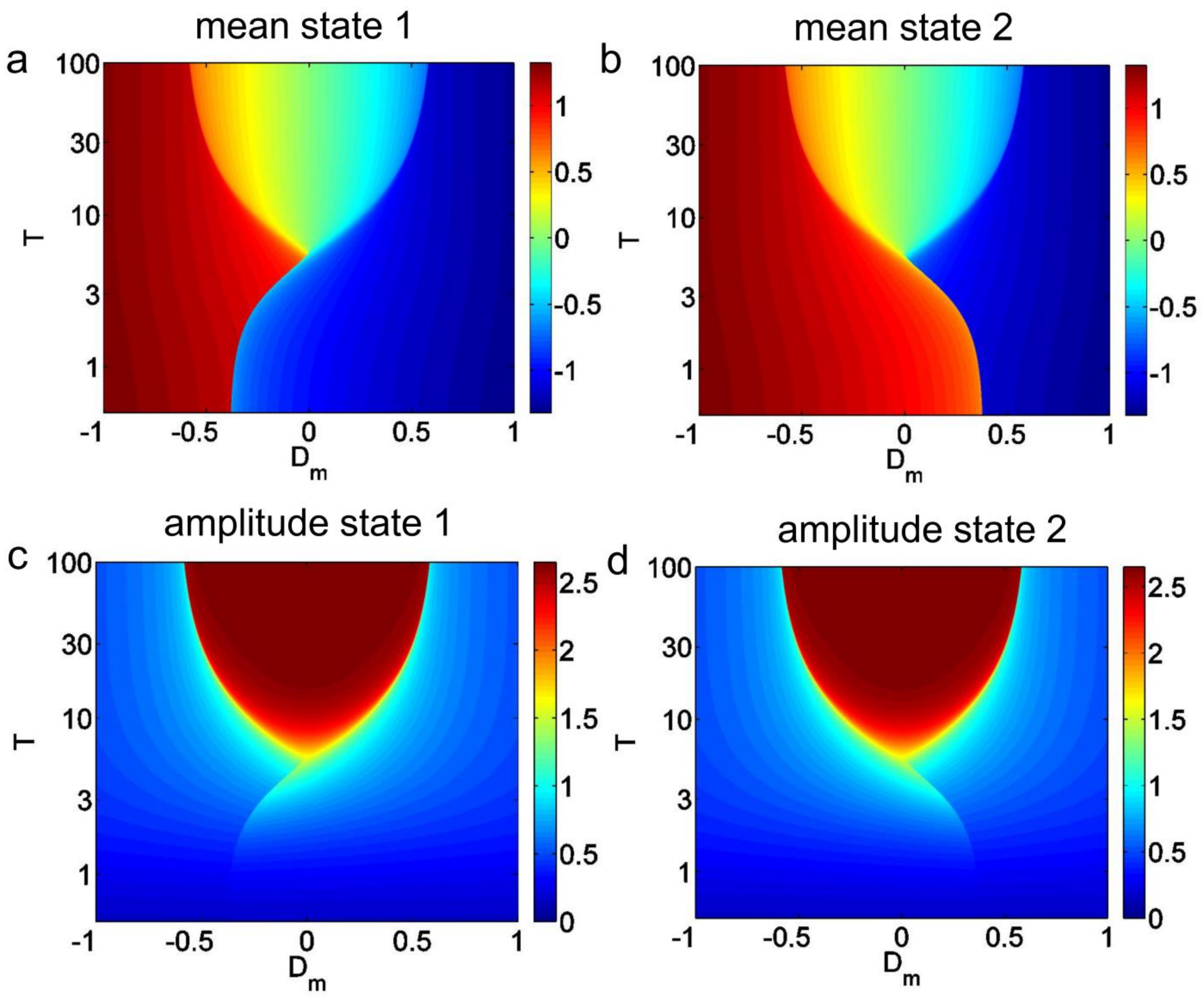

Fig. S2. As Fig. S1 but for driver period T versus mean D_m.

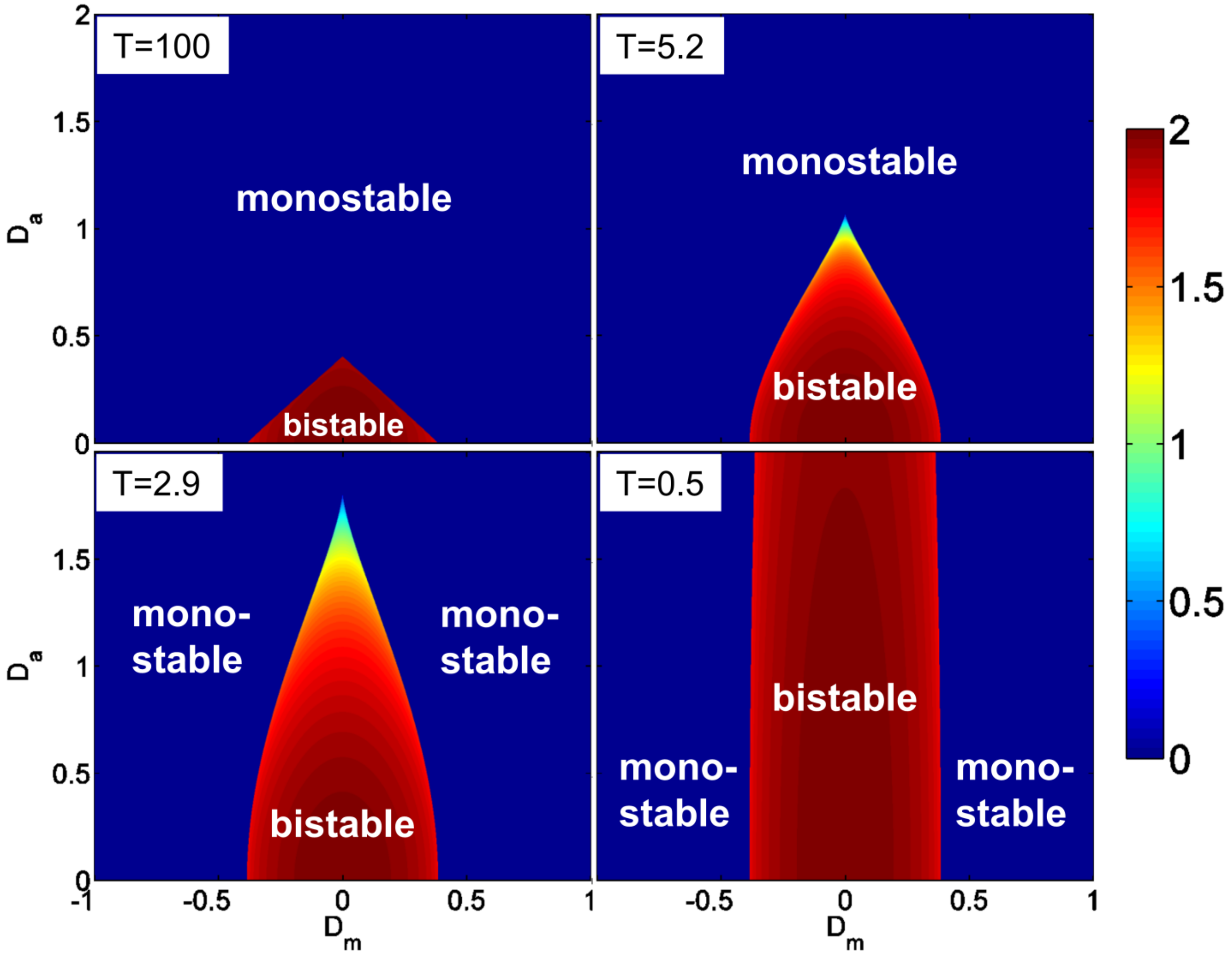

Fig. S3. Dependence of the example system on initial conditions for different parameters D_m (horizontal axis), amplitude D_a (vertical axis) and period T (subfigures). The color shows the time mean of the absolute difference between the system's solutions. This difference is 0 (blue) if there is only one solution (monostable regime) and > 0 if the system is bistable; the boundaries between these regimes hence consist in bifurcation points.

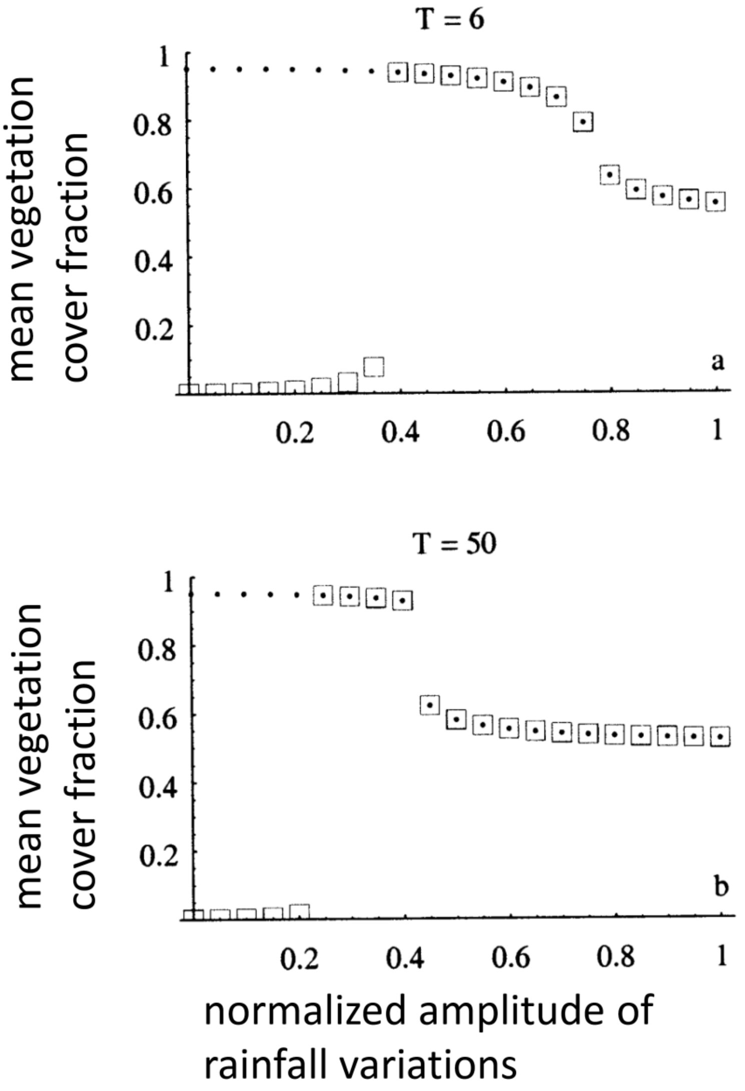

Fig. S4. Modified Fig. 10 from Zeng et al.[87]. Results from a conceptual dynamical model of atmosphere-vegetation interaction showing two stable states for a range of parameters. Precipitation is fluctuating with a given amplitude (horizontal axis) and a given period T (top and bottom figures are for different T). The model then calculates a vegetation coverage whose time mean is shown on the vertical axis. As in Fig. S1-S3, two stable states exist for small amplitude forcing, and the transition to the remaining state is more gradual for fast forcing (low T) than slow forcing (large T). ©American Meteorological Society. Used with permission.
